# Recovering Pressure-Independent Dilute-Gas Thermal Conductivity from Molecular Dynamics: Green–Kubo Sampling Sensitivity and NEMD Finite-Size Effects

**DOI:** 10.3390/nano16191241

**Published:** 2026-10-01

**Authors:** Matthew Riggs, Atal Bhowmik, Murat Barisik

**Affiliations:** 1 Mechanical Engineering Department, University of Tennessee at Chattanooga, Chattanooga, TN 37403, USA; ldd912@mocs.utc.edu (M.R.); zhf342@mocs.utc.edu (A.B.); 2 University of Tennessee at Chattanooga (UTC) Nanoengineering Center, University of Tennessee at Chattanooga, Chattanooga, TN 37403, USA

**Keywords:** ideal gas, thermal conductivity, molecular dynamics, Green–Kubo method, nonequilibrium molecular dynamics, statistical convergence

## Abstract

Gas thermal conductivity is important in many engineering systems, but reliable data are not available for every gas, mixture, temperature, and pressure. Molecular dynamics can help fill this gap by predicting transport directly from molecular motion. However, gas simulations face time- and length-scale limitations that are different from those commonly encountered in liquids and solids. These limitations are not always recognized, and standard molecular dynamics tools are often used without checking whether their settings are suitable for dilute gases. This study uses the pressure-independent thermal conductivity of dilute argon as a benchmark for evaluating equilibrium and nonequilibrium molecular dynamics methods. At fixed temperature, changing pressure changes the mean free path and collision time even though the bulk thermal conductivity remains constant. Green–Kubo calculations at 300 K and 1 atm produced values from 0.009 to 0.022 W m^−1^ K^−1^ as the sampling interval and correlation length were varied, compared with the expected value of about 0.0175 W m^−1^ K^−1^. Some settings reproduced the expected value without showing convergence, demonstrating the risk of using Green–Kubo as a black-box calculation. NEMD simulations from 0.2 to 1.0 atm showed that the apparent conductivity increased with longitudinal system size and approached similar asymptotic values across pressure. The results recover pressure-independent gas thermal conductivity while showing that decreasing pressure increases the molecular length and time scales relevant to MD simulations. Reliable gas-property prediction therefore requires direct checks of Green–Kubo time scales and NEMD system-size dependence.

## 1. Introduction

The bulk thermal conductivity of a gas has a simple but surprising property: for an ordinary gas in the ideal-gas transport regime, it is independent of pressure or density at fixed temperature. This is a bulk-property statement. As pressure decreases, fewer molecules are available to carry energy, which would be expected to reduce heat conduction. However, the molecular mean free path increases, so each molecule carries energy over a longer distance between collisions. These two effects balance each other, causing the leading pressure dependence of thermal conductivity to cancel [1,2]. This pressure independence applies as long as the transport being measured represents bulk gas behavior; apparent size effects can develop when a characteristic system length becomes comparable to the molecular mean free path [3,4,5,6,7].

Reliable gas thermal conductivity data are needed in heat exchangers, thermal insulation, aerospace systems, gas processing, energy technologies, and computational fluid dynamics. For common gases, these values are usually obtained from experiments, reference databases, or empirical correlations [8,9,10]. However, available data do not cover every gas mixture, temperature range, pressure range, or newly proposed working fluid. This creates a need for predictive methods that can extend property knowledge beyond existing measurements. Molecular dynamics simulations are attractive for this purpose because they calculate transport directly from molecular motion, collisions, and intermolecular interactions rather than relying only on fitted macroscopic correlations [11,12,13,14].

Two general molecular dynamics approaches are commonly used to calculate thermal conductivity. In nonequilibrium molecular dynamics (NEMD), a temperature gradient or heat flux is imposed, and the conductivity is obtained from the resulting heat flow using Fourier’s law [15,16,17]. In equilibrium molecular dynamics (EMD), the system is simulated at equilibrium, and the conductivity is obtained from spontaneous heat-flux fluctuations through the Green–Kubo formalism [17,18,19]. Both approaches are well established and have been applied to many classes of materials, including solids, liquids, and gases [11,16,20,21,22,23]. The choice between them is usually practical rather than absolute: EMD avoids imposing an external thermal gradient, while NEMD provides a direct heat-transfer response that can be inspected through the temperature profile and heat flux [24,25].

Despite this strong theoretical foundation, MD thermal-conductivity calculations are not automatic property evaluations. In EMD, the heat-flux autocorrelation function must be sampled at an appropriate frequency and integrated over a suitable correlation time [26,27,28]. A correlation window that is too short misses relevant heat-flux memory, while a window that is too long can allow long-time noise to dominate the integral [29,30,31]. In NEMD, the imposed heat flux or temperature difference must be strong enough to overcome thermal fluctuations but weak enough to remain in the linear-response regime [16,20,32]. The simulation domain and averaging time must also be sufficient for the calculated response to represent the intended bulk transport property [3,4,5,6,7,33].

These practical issues are especially important for gases because the molecular mean free path and collision time increase strongly as pressure decreases, even when the true bulk thermal conductivity should remain constant [1,2]. This creates a clear risk in both EMD and NEMD calculations. In EMD, a Green–Kubo calculation can produce a numerical conductivity even when the heat-flux autocorrelation is not properly resolved or converged [25,29,30,34]. In NEMD, a simulation can produce a temperature gradient and an apparent conductivity even when the system length, averaging time, or applied heat flux are not sufficient to represent bulk gas transport [3,4,5,33]. In both cases, the result may look like a valid thermal conductivity calculation, but it may be an artifact of the simulation setup rather than a property of the gas. This is a particular concern when users transfer settings from benchmark examples or previous studies without checking whether those settings remain appropriate for a gas-phase system [35,36,37].

This study uses the known pressure-independent conductivity of dilute argon as a test of molecular dynamics methodology. The Green–Kubo calculations examine how standard LAMMPS sampling and correlation choices can produce apparently reasonable but parameter-dependent conductivity values. The NEMD calculations examine how a finite simulation length can produce an apparent conductivity that has not yet reached the bulk limit. Together, these tests demonstrate why MD conductivity calculations must be examined for both time-sampling and system-length convergence before being accepted as material-property data.

## 2. Kinetic-Theory Background for Gas Thermal Conductivity

The pressure independence of dilute-gas thermal conductivity follows from kinetic-theory scaling:

(1) $$\begin{array}{c} k∼nc_{v}\overset{ˉ}{v}\lambda , \end{array}$$

where *k* is thermal conductivity, *n* is the number density, *c_v_* is the molecular heat capacity, $\overset{ˉ}{v}$ is the characteristic molecular speed, and *λ* is the mean free path. For a dilute gas, the mean distance traveled between binary collisions can be calculated from

(2) $$\begin{array}{c} \lambda =\frac{1}{\sqrt{2}\pi d^{2}n} \end{array}$$

where *d* is the molecular collision diameter. Substitution of Equation (2) into Equation (1) gives

(3) $$\begin{array}{c} k=\frac{c_{v}\overset{ˉ}{v}}{\sqrt{2}\pi d^{2}} \end{array}$$

The number density therefore cancels from the leading-order relation. At fixed temperature, *c_v_*, $\overset{ˉ}{v}$ and the molecular collision properties remain approximately constant for a monatomic gas. The bulk thermal conductivity is consequently independent of pressure. More complete kinetic-theory treatments introduce collision integrals and numerical factors but retain the same dilute-gas behavior [1,2].

This cancellation applies to *k* but not to the molecular length and time scales. Using the ideal-gas relation, the mean free path becomes

(4) $$\begin{array}{c} \lambda =\frac{k_{B}T}{\sqrt{2}\pi d^{2}P} \end{array}$$

where *k_B_* is the Boltzmann constant, T is temperature, and *P* is pressure. Thus, lowering the pressure increases the mean free path, even though the bulk thermal conductivity remains unchanged. The same pressure dependence appears in the molecular collision time. The corresponding mean collision time is

(5) $$\begin{array}{c} \tau =\frac{\lambda }{\bar{\nu }} \end{array}$$

while the molecular speed is

(6) $$\begin{array}{c} \bar{\nu }=\sqrt{\frac{8k_{b}T}{\mathrm{m\pi}}} \end{array}$$

At fixed temperature, the characteristic molecular speed remains constant, while both the mean free path and collision time increase as pressure decreases. Therefore, pressure independence of the bulk thermal conductivity does not imply pressure-independent MD requirements. Longer collision times affect the sampling required in Green–Kubo calculations, while larger mean free paths affect the simulation length required in NEMD calculations. These two consequences are examined in Section 4.1 and Section 4.2.

## 3. Molecular Dynamics Methods

### 3.1. Molecular Model and Simulation Conditions

Molecular dynamics simulations were performed using the Large-scale Atomic/Molecular Massively Parallel Simulator (LAMMPS), version 11 February 2026 (Sandia National laboratories, Albuquerque, NM, USA) [38]. Argon atoms were modeled using the Lennard–Jones potential,

(7) $$\begin{array}{c} \phi_{0}\left(r\right)=4\epsilon \left[{\left(\frac{\sigma }{r}\right)}^{12}-{\left(\frac{\sigma }{r}\right)}^{6}\right] \end{array}$$

where *r* is the distance between atoms, *ε* is the potential-well depth, and *σ* is the molecular diameter. The Lennard–Jones parameters for argon were *σ* = 3.405 Å and *ε*/*k_B_* = 119.8 K [39]. The interaction potential was truncated at 10 Å, treating the interaction energy as zero for interactions of particles that are farther than 10 Å. Electrostatic interactions were not included because argon was represented as a neutral monatomic gas. Periodic boundary conditions were applied in all three directions to represent a periodically replicated bulk system. Initial configurations were energy-minimized using the conjugate-gradient method to remove unfavorable atomic overlaps. The equations of motion were integrated using the velocity-Verlet algorithm with a timestep of 1 fs. All simulations were performed at 300 K. The investigated pressures were 1.0, 0.8, 0.6, 0.4, and 0.2 atm. For each pressure, the number of atoms and simulation volume were selected to reproduce the corresponding number density.

### 3.2. Equilibrium Molecular Dynamics (EMD) and Green–Kubo Formulation

In equilibrium molecular dynamics, thermal conductivity is calculated from spontaneous fluctuations of the microscopic heat flux. For a system governed by pairwise interactions, the microscopic heat flux can be expressed as

(8) $$\begin{array}{c} J(t)=\frac{1}{V}\left[\sum_{i}e_{i}v_{i}-\sum_{i}S_{i}v_{i}\right] \end{array}$$

where *V* is the system volume, *e_i_* is the total kinetic and potential energy assigned to atom *i*, *ν_i_* is its velocity, and *S_i_* is its per-atom stress tensor. The first term represents energy transported by molecular motion, while the second represents the contribution associated with intermolecular interactions.

The heat-flux autocorrelation function is defined as

(9) $$\begin{array}{c} C\left(J\left(t\right)\right)=\langle J\left(0\right)\cdot J(t)\rangle \end{array}$$

and measures the extent to which the instantaneous heat flux retains memory of its initial value. Molecular motion and collisions progressively redistribute energy, causing the autocorrelation function to decay and eventually fluctuate around zero.

The Green–Kubo relation connects the autocorrelation function to the thermal conductivity:

(10) $$\begin{array}{c} k=\frac{V}{3k_{B}T^{2}}\int_{0}^{\infty }\langle J\left(0\right)\cdot J(t)\rangle \mathrm{dt} \end{array}$$

where *k_B_* is the Boltzmann constant and *T* is the system temperature. The factor of three accounts for the three equivalent spatial directions of the isotropic argon system.

The Einstein–Helfand formalism provides an alternative equilibrium approach based on the long-time evolution of an energy-displacement moment. However, its implementation for an interacting system with periodic boundaries requires additional corrections and extensive trajectory post-processing. It was therefore not used in the present study, which focused on the Green–Kubo approach.

In LAMMPS, the atomic kinetic energy, potential energy, and per-atom stress contributions were used to calculate the microscopic heat flux. The fix ave/correlate command was then used to calculate the heat-flux autocorrelation function over the selected correlation interval. The Green–Kubo integral was evaluated numerically using the trapezoidal rule. In the LAMMPS fix ave/correlate implementation, three parameters control the construction of the heat-flux autocorrelation function. The sampling interval s (Nevery) specifies the number of MD timesteps between successive heat-flux samples. The correlation length *p* (Nrepeat) specifies the number of correlation points, while d (Nfreq) specifies how frequently the averaged correlation function is reported. In the present calculations, d was set equal to sp. These parameters follow the standard LAMMPS Green–Kubo implementation.

For an MD timestep Δ*t*, the physical sampling interval is *t_s_* = *s*Δ*t*, while the maximum correlation window is

(11) $$\begin{array}{c} t_{w}=\left(p-1\right)s\Delta t\approx \mathrm{ps}\Delta t \end{array}$$

Increasing *p* allows for the heat-flux autocorrelation function to be followed over a longer lag time. However, for a fixed production duration *t_d_*, it also reduces the nominal number of complete, nonoverlapping correlation windows available within the trajectory:

(12) $$\begin{array}{c} N_{w}\approx \frac{t_{d}}{t_{w}} \end{array}$$

Because ave running accumulates correlations using multiple and overlapping time origins, *N_w_* is not the number of statistically independent samples. It is used here only to illustrate the tradeoff between extending the correlation window and retaining sufficient trajectory data for statistical averaging.

With the timestep of 1 fs used here, s = 90 corresponds to a physical sampling interval of 90 fs. The combinations s = 90, *p* = 3000 and s = 90, *p* = 7000 correspond to maximum correlation windows of approximately 270 and 630 ps, respectively. A correlation window that is too short can truncate physically relevant heat-flux correlations, whereas extending the window beyond the meaningful decay can add long-time statistical noise to the Green–Kubo integral. The result must therefore be tested for stability with respect to the sampling, correlation, and integration choices rather than accepted from a single parameter combination.

Alternative approaches, including autocorrelation-tail fitting and adaptive Green–Kubo integration, have been proposed to reduce the effect of long-time noise [26,27,30]. The present work instead examines the sensitivity of the standard trapezoidal Green–Kubo implementation because this is the procedure commonly transferred from LAMMPS examples to new systems.

For the equilibrium calculations, each system was equilibrated for 2 ns in the canonical (NVT) ensemble at 300 K. This was followed by a 480 ns production calculation in the microcanonical (NVE) ensemble, during which the microscopic heat flux was recorded. The correlation length and integration selection were varied to determine their effects on the calculated conductivity. The combinations of these parameters were chosen to consider four comparisons. First, s was held constant while *p* was varied to evaluate the effect of the correlation-window length. Second, *p* was held constant, while s was varied to evaluate the effect of sampling frequency. Third, different combinations of s and *p* were examined while maintaining an approximately constant total correlation-window duration, allowing for the determination of dependence on the individual selection of s and p. Finally, the length of the cubic simulations was varied to assess the influence of length on the calculated conductivity.

The heat-flux quantities obtained from LAMMPS were converted using the required simulation-volume and unit-conversion factors, and the final conductivities were reported in W m^−1^ K^−1^. LAMMPS returns a numerical Green–Kubo conductivity for any valid selection of these parameters; therefore, the existence of a numerical value, or even agreement with a known reference value, does not demonstrate convergence. The resulting parameter sensitivity is examined in Section 4.1.

### 3.3. Nonequilibrium Molecular Dynamics (NEMD) Formulation

In nonequilibrium molecular dynamics, heat transfer is imposed directly and the thermal conductivity is calculated from the resulting temperature gradient using Fourier’s law:

(13) $$\begin{array}{c} {q^{\prime \prime }}_{z}=\;-k\left(\frac{\mathrm{dT}}{\mathrm{dz}}\right) \end{array}$$

where (*q″_z_*) is the heat flux in the (*z*)-direction and (*dT*/*dz*) is the corresponding temperature gradient.

**Figure 1:**
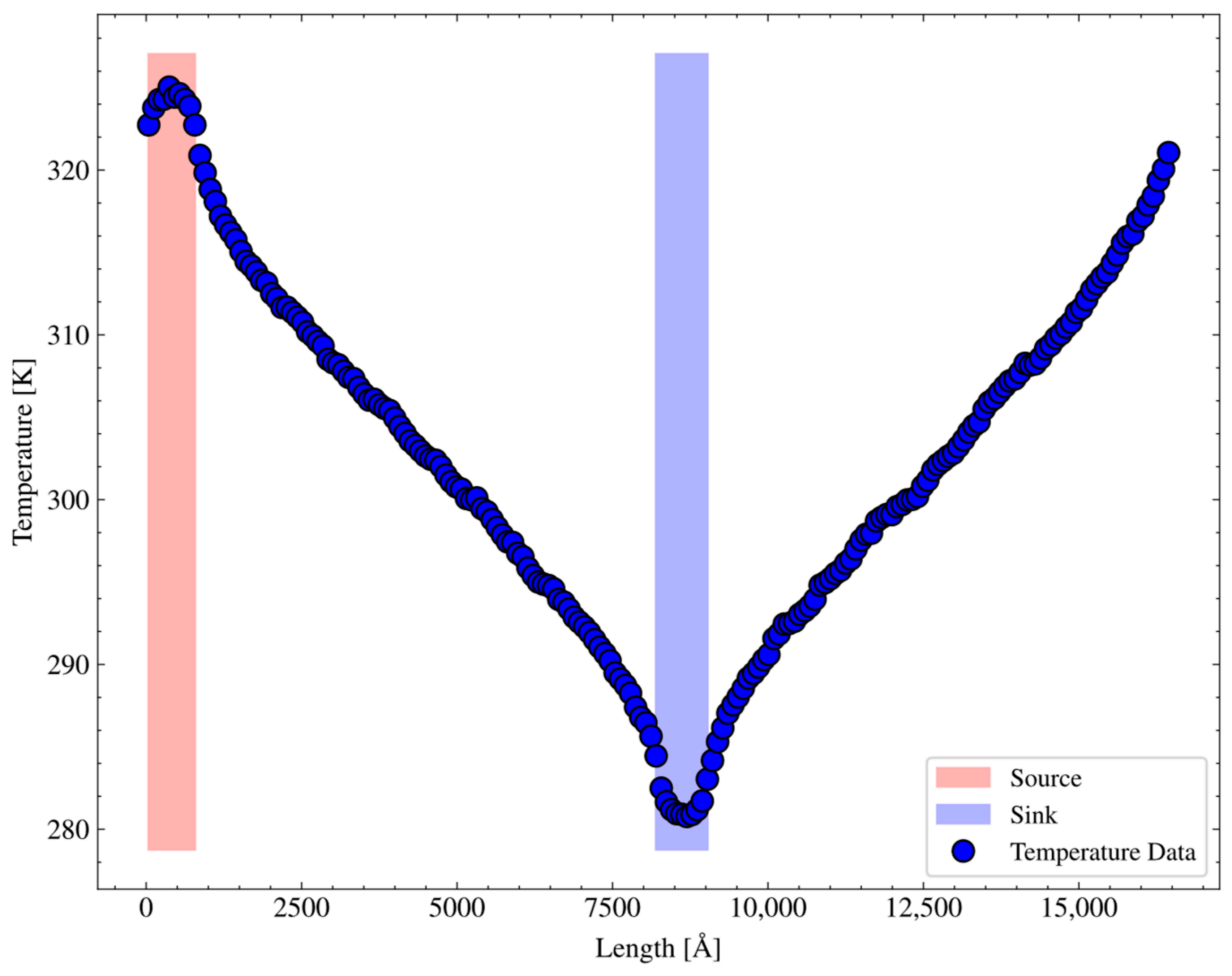
The temperature profile for the 1 atm, 300 K, L_z_* = 24 case.

The present simulations used the enhanced heat-exchange method developed by Wirnsberger et al. [40]. Equal amounts of energy were added to the heat-source region and removed from the heat-sink region. The imposed energy-transfer rate was $\dot{Q}$ = 0.01 eV/ps. This value was selected by examining the resulting temperature profiles and confirming that two well-defined linear regions developed between the source and sink, as illustrated in Figure 1 for the 1 atm, 300 K, L_z_* = 24 case.

The localized nonlinearities within the source and sink bins—arising from direct energy addition and removal—were excluded from the gradient calculation, and Fourier’s law was applied only to the linear portions of the profile to obtain the thermal conductivity.

**Figure 2:**
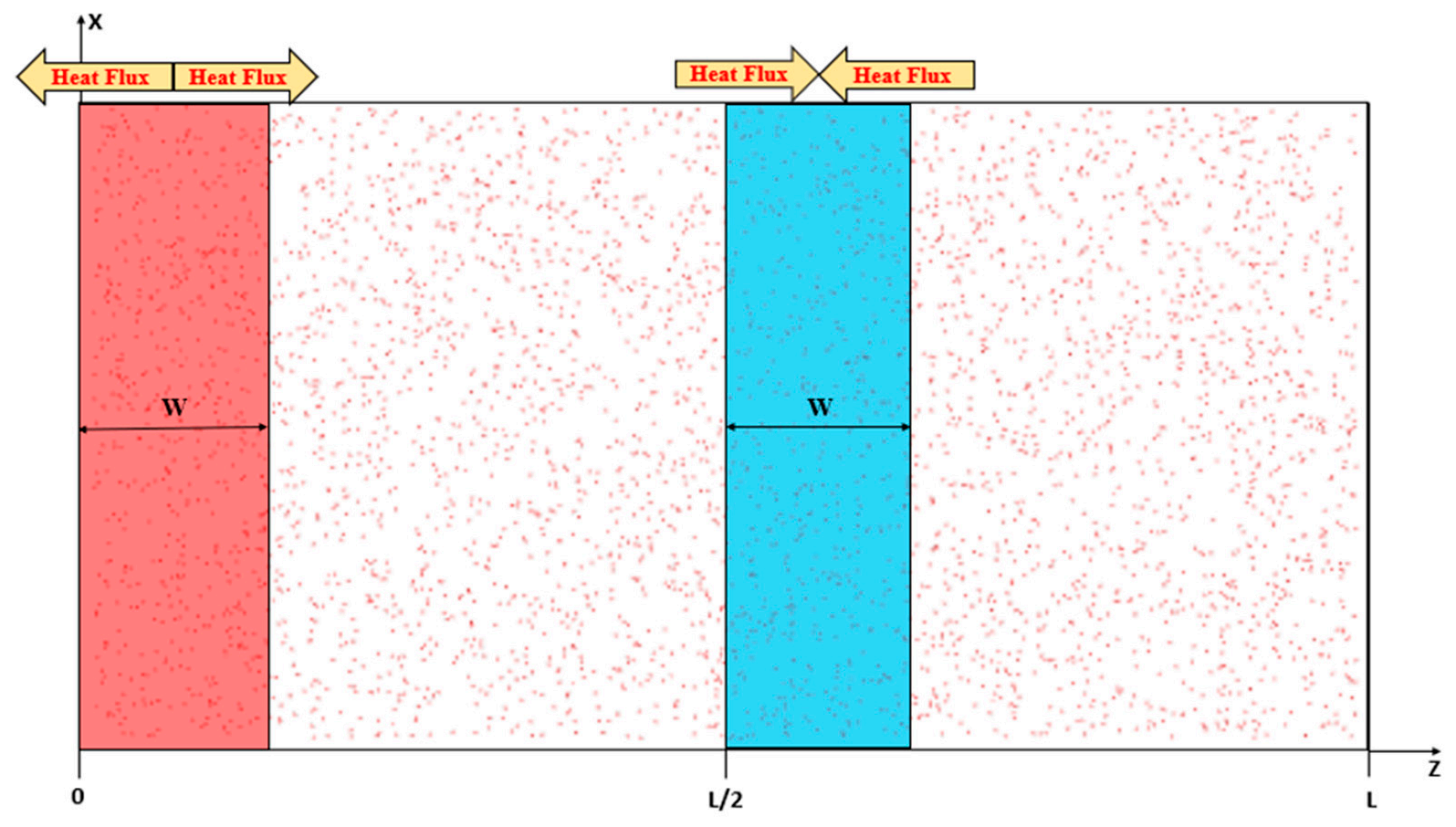
The NEMD simulation configuration showing the heat-source (red) and heat-sink (blue) regions and the two heat-transfer paths created by periodic boundary conditions. Representative short and long simulation domains are shown.

The heat flux established a temperature gradient while limiting the energy drift associated with the original heat-exchange algorithm. The simulation box was divided into 200 bins along the (z)-direction. Ten bins were assigned to the heat-source region and ten bins to the heat-sink region. Periodic boundary conditions created two equal heat-transfer paths between the source and sink, as illustrated in Figure 2.

Because the imposed energy flowed from the source to the sink in two directions, one-half of the total energy-transfer rate contributed to each temperature gradient. The thermal conductivity was therefore calculated as

(14) $$\begin{array}{c} k=\frac{\dot{Q}}{2A\langle \left|\frac{\mathrm{dT}}{\mathrm{dz}}\right|\rangle } \end{array}$$

where ($\dot{Q}$) is the total imposed energy-transfer rate, (*A*) is the cross-sectional area, and ($\langle \left|\frac{\mathrm{dT}}{\mathrm{dz}}\right|\rangle$) is the average magnitude of the temperature gradient between the source and sink. The source and sink bins were excluded from the temperature-gradient calculation. Separate linear fits were applied to the two source-to-sink regions, and the average magnitude of the two fitted slopes was used in Equation (14). The quantity ($\dot{Q}$) in Equation (14) is the imposed energy-transfer rate used in NEMD. It is distinct from the instantaneous microscopic heat-flux vector (J(t)) used in the Green–Kubo calculation. Each NEMD system was equilibrated for 2 ns using the canonical (NVT) ensemble. The production simulation was then performed for 500 ns using microcanonical (NVE) time integration together with the enhanced heat-exchange algorithm. The temperature profile was averaged after the system reached a steady nonequilibrium state. To determine when the nonequilibrium state occurs, the temperature profile at each window is examined, and the change in temperature between each window at every spatial point in the heat-flux direction is determined.

(15) $$\begin{array}{c} \Delta T_{i}\left(L_{Z_{j}}\right)=T_{i+1}\left(L_{Z_{j}}\right)-T_{i}\left(L_{Z_{j}}\right) \end{array}$$

Using the change in the temperature profile between each window, the Root Mean Square (RMS) for each window is

(16) $$\begin{array}{c} \Delta T_{\mathrm{RM}S_{i}}=\sqrt{\frac{1}{N_{L_{Z}}}\sum_{j=1}^{N_{L_{Z}}}{\left[\Delta T_{i}(L_{Z_{i}})\right]}^{2}} \end{array}$$

where *i* is every window and *j* is every spatial point in each window. The RMS is used since it determines how much the temperature profile changes, on average, from one window to the next without allowing cancellation of negative and positive temperature changes. The window number vs. RMS is plotted for examination of the RMS to determine where the RMS plateaus. This allows for the determination of the first point in averaging of the windows for the temperature profiles. The RMS profile for the 1 atm, 300 K, and L_z_* = 24 case is shown below.

**Figure 3:**
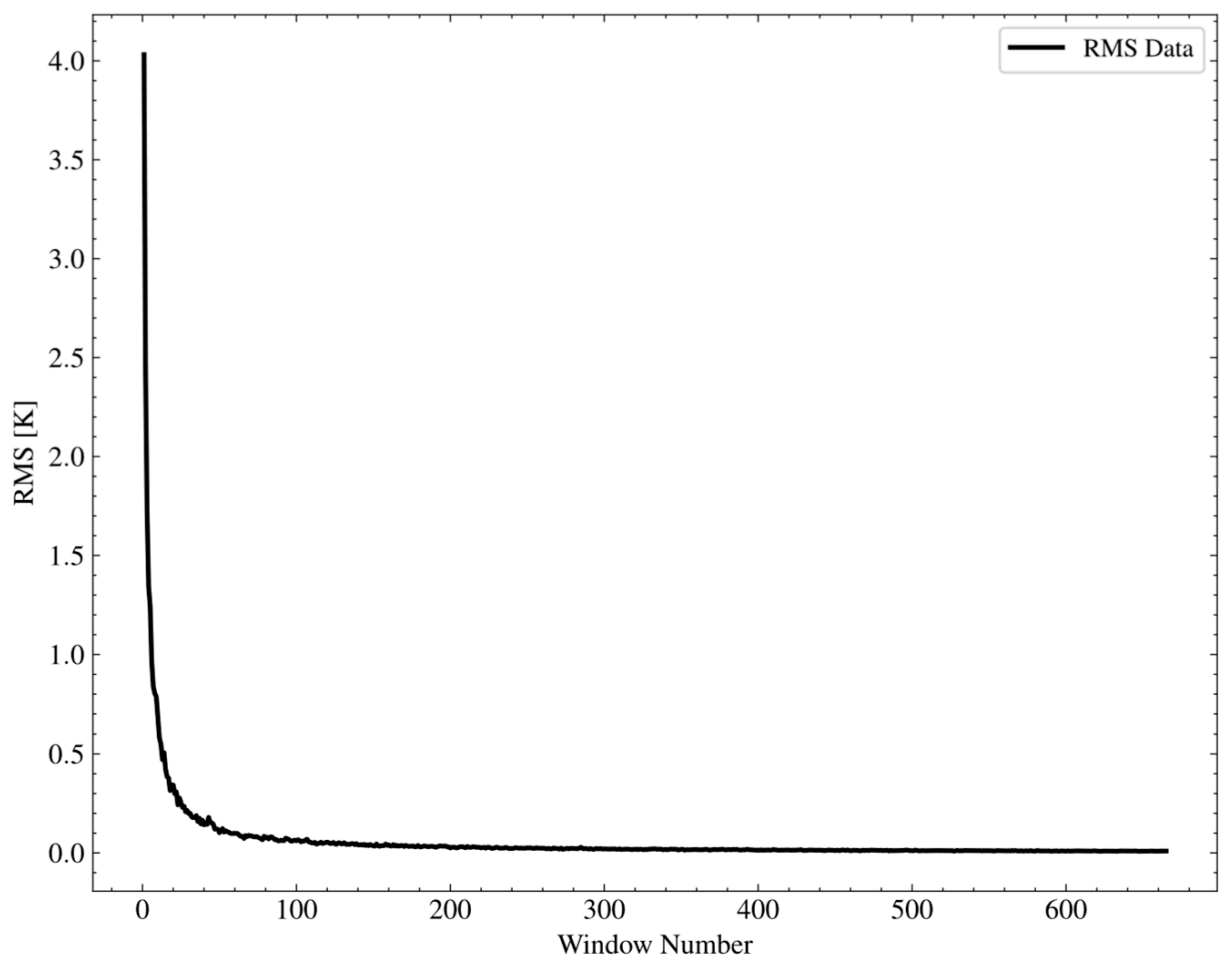
The Root Mean Square (RMS) for every window number for the determination of the steady nonequilibrium temperature profile for NEMD 1 atm, 300 K, L_z_* = 24.

It is seen in Figure 3 that the RMS plateaus around the 200th window. Using the 200th window to the last observed data point, the thermal conductivity is determined to be 16.27 mW/m/k.

**Figure 4:**
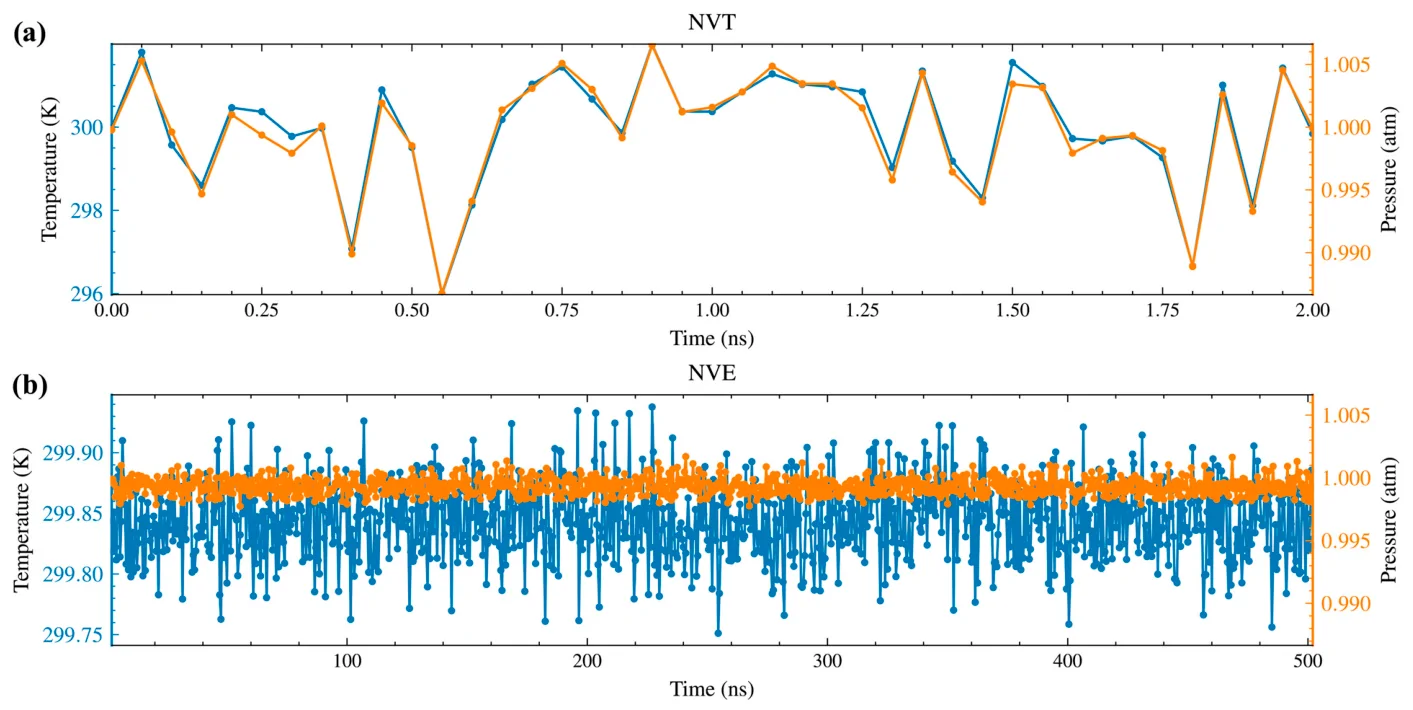
Temperature (primary *y*-axis) and pressure (secondary *y*-axis) histories for the 300 K and 1 atm argon system during (**a**) NVT equilibration and (**b**) NEMD production using NVE time integration with enhanced heat exchange.

Figure 4 presents the temperature and pressure histories for the 300 K and 1 atm case. Panel (a) corresponds to the NVT equilibration stage, and panel (b) corresponds to the NEMD production stage.

The production stage is not an equilibrium NVE simulation because energy was continuously added to the source region and removed from the sink region. NVE refers only to the time-integration procedure used together with the enhanced heat-exchange algorithm.

## 4. Results and Discussion

### 4.1. Green–Kubo Results and Sensitivity to Sampling and Integration Parameters

The Green–Kubo calculations were first examined to determine how the selected sampling and integration parameters affected the calculated thermal conductivity of dilute argon. The Green–Kubo thermal conductivity of dilute argon gas at 300 K and 1 atmosphere showed high sensitivity to the correlation-window time. Table 1 summarizes the conductivity values obtained using different sampling intervals, correlation lengths, correlation-window times, and simulation sizes using three independent simulations for determination of the mean and 95% confidence interval for conductivity values. The kinetic-theory value at 300 K was approximately 0.0175 W m^−1^ K^−1^.

The directional conductivities varied from approximately 0.009 to 0.022 W m^−1^ K^−1^, while the kinetic-theory value for the investigated state was approximately 0.0175 W m^−1^ K^−1^. Averaged over the three normalized characteristic lengths, the calculated conductivity increased from 0.00998 W m^−1^ K^−1^ at T_w_ = 0.27 ns to 0.01759 W m^−1^ K^−1^ at T_w_ = 1.08 ns, corresponding to deviations of approximately −43% and 0.51%, respectively, from the kinetic-theory value. If the correlation window extended further to 2.5–3.0 ns, the estimate increased to approximately 0.0182–0.0189 W m^−1^ K^−1^, or 4–8% above the theoretical value. Different combinations of sampling interval and correlation length produced nearly the same conductivity when they resulted in similar correlation-window times T_w_. For example, T_w_ = 0.60 and 0.63 ns produced similar conductivities, as did T_w_ = 2.50 and 2.52 ns. This indicates that within the tested range, the correlation-window duration rather than s or *p* individually controlled the main variation in the Green–Kubo conductivity. As T_w_ increased, the conductivity initially approached the expected value. At T_w_ = 1.08 ns, the average conductivity was close to the reference value. However, extending the correlation window further increased the scatter among the directional conductivities and shifted the average conductivity above the reference value. Thus, increasing the correlation window did not produce a simple monotonic convergence toward the expected conductivity.

**Figure 5:**
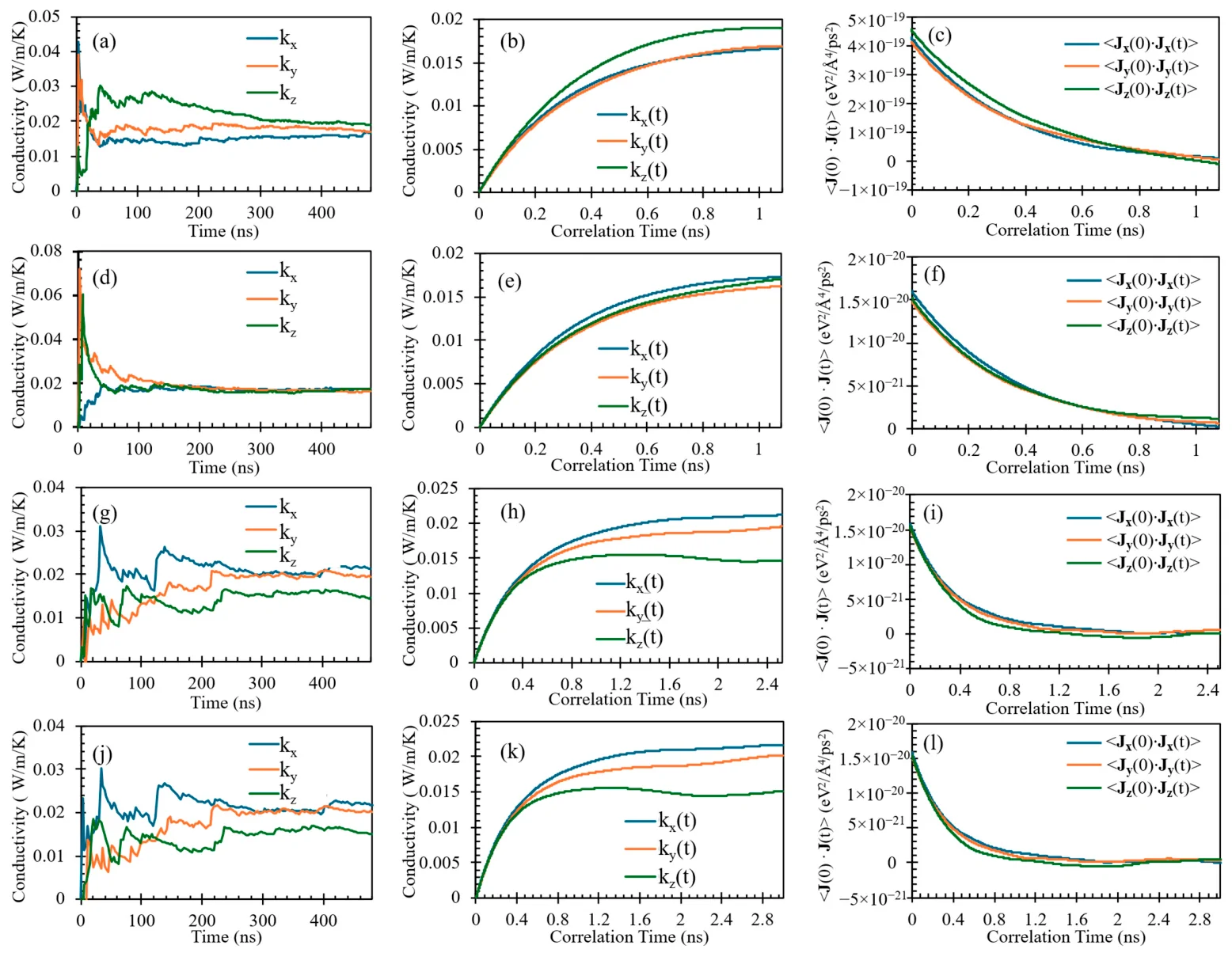
Green–Kubo thermal-conductivity convergence for dilute argon at 300 K and 1 atm. Panels (**a**–**c**) correspond to s = 360, *p* = 3000, L* = 1; (**d**–**f**) to s = 360, *p* = 3000, L* = 3; (**g**–**i**) to s = 360, *p* = 7000, L* = 3; and (**j**–**l**) to s = 1000, *p* = 3000, L* = 3. The left panels show the evolution of the directional conductivity components during the 480 ns production simulation, the middle panels show corresponding running Green–Kubo integrals as a function of correlation lag time, and the right panels show the HFACF as a function of correlation time. The visible HFACF magnitude differences between panel (**c**) and panels (**f**,**i**,**l**) arise from the inversely proportional relationship (~1/V) of HFACF and volume; consequently, the profiles for L* = 3 are roughly 1/27th of the height of the L* = 1 curve due to the 27-fold difference in volume.

Figure 5 shows three separate convergence checks. The left panels show whether the calculated conductivity becomes stable as more production data are accumulated; the middle panels show whether the running Green–Kubo integral reaches a plateau with correlation time, while the right panels show whether the HFACF has reached zero and accumulated noise. For the T_w_ = 1.08 ns cases, the conductivity is close to the expected value even though the running integrals are still changing. At longer correlation windows, the directional conductivities separate further. Thus, agreement with the expected conductivity at one selected correlation window does not demonstrate Green–Kubo convergence.

**Figure 6:**
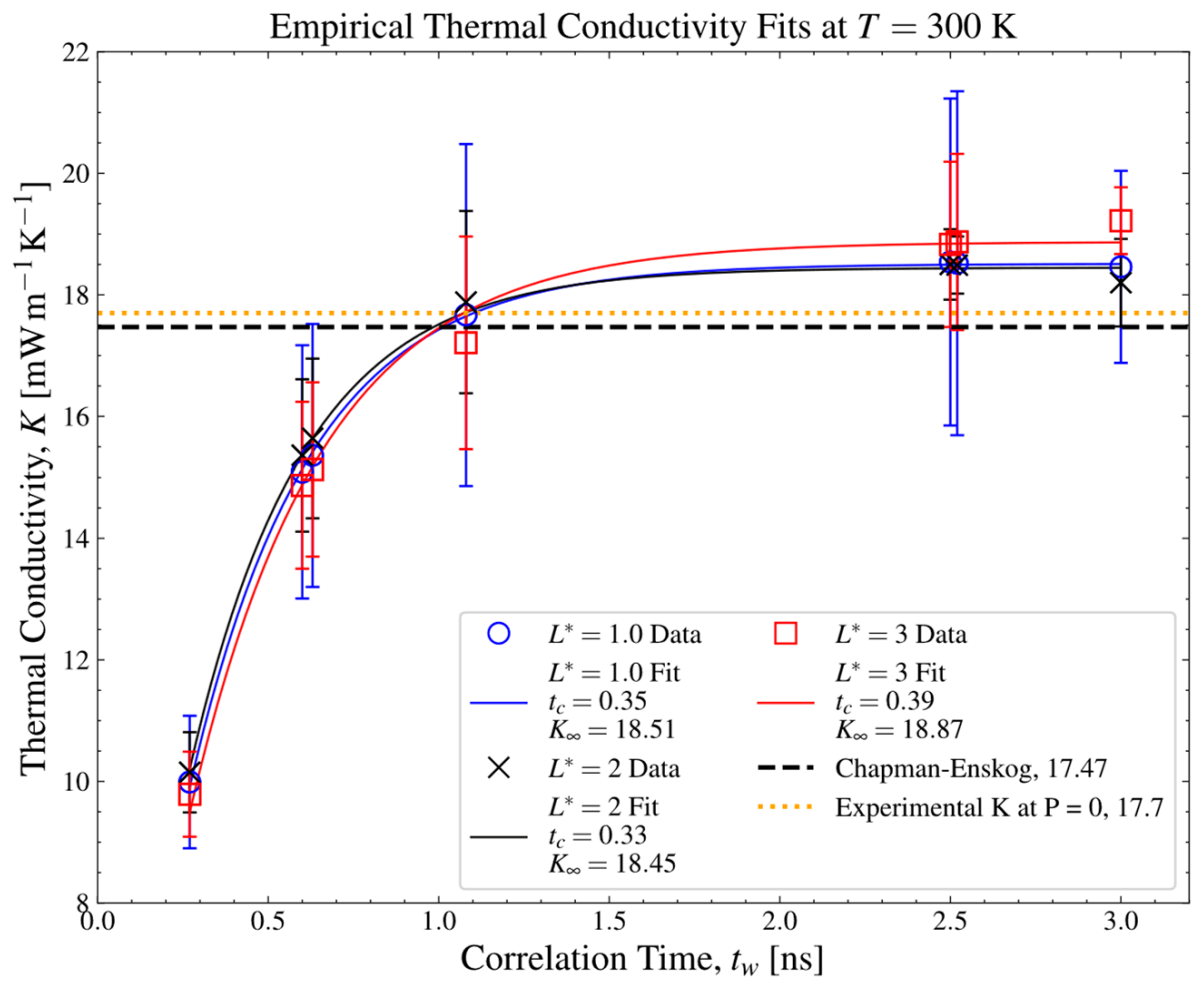
Green–Kubo thermal conductivity as a function of correlation time for 1 atm. Symbols represent the simulation results, and lines represent the empirical fits.

Finite-time convergence of the calculated conductivity can be represented using a characteristic–time relation [26],

(17) $$\begin{array}{c} k\left(t\right)=k_{\infty }\left[1-e^{-\frac{t}{t_{c}}}\right] \end{array}$$

where *t* is the observation or correlation time, *t_c_* is a characteristic relaxation time, and *k*_∞_ is the long-time conductivity. This relation emphasizes that a conductivity obtained at one selected correlation time does not establish convergence unless the sampled time is sufficiently long relative to the characteristic dynamics of the system. In the present work, Equation (17) was used only to describe the observed dependence on correlation-window time. The fitted asymptote was not taken as proof of Green–Kubo convergence. Figure 6 presents the Green–Kubo thermal-conductivity values using characteristic–time relations determined from the values obtained in Table 1.

Under the simulation durations and parameter ranges investigated here, a clearly parameter-independent Green–Kubo conductivity was not recovered. This does not demonstrate that the Green–Kubo formalism is fundamentally unsuitable for dilute gases. It shows that a plausible conductivity can be obtained without demonstrating statistical convergence, particularly when the heat-flux correlations are weak and noisy.

The Green–Kubo results confirm the suitability of dilute-gas thermal conductivity as an extremely demanding test for molecular simulations. It is possible to obtain Green–Kubo results consistent with the expected thermal conductivity of argon, but the accuracy of the results obtained will be highly dependent on the duration of the integration window and the length of statistical averaging. Too short integration windows will fail to capture important correlations, while too long windows might introduce statistical noise into the system, leading to biased scatter and drift upwards. Therefore, the issue is not the validity of the Green–Kubo formalism, but the selection of a statistically justified correlation window. The NEMD calculations examine the separate question of whether the simulation length is sufficient relative to the molecular mean free path.

### 4.2. Mean-Free-Path-Scaled System-Size Dependence of the NEMD Conductivity

The NEMD simulations were used to test how the calculated conductivity changed as the simulation dimensions increased relative to the molecular mean free path. The normalized dimension was defined as

(18) $$\begin{array}{c} L_{i}^{*}=\frac{L_{i}}{\lambda } \end{array}$$

where (*L_i_*) is the simulation-box dimension in direction (*i*) and (*λ*) is the mean free path at the corresponding temperature and pressure. The mean free path was calculated using the kinetic-theory relation introduced in Section 2. The transverse dimensions were fixed at Lₓ* = L_y_* = 0.5, while the longitudinal dimension (L_z_*) was varied from 2 to 24. The simulation characteristics are shown in Table 2.

This allowed for the dependence of the calculated conductivity on the longitudinal simulation size to be examined relative to the molecular mean free path.

Because the mean free path increases as pressure decreases, the physical dimensions corresponding to a given normalized length were larger at lower pressure. Thus, the simulations tested equivalent dimension-to-mean-free-path ratios rather than identical physical box lengths at every pressure. The conductivity data at each pressure were fitted using the empirical relation

(19) $$\begin{array}{c} k\left(L\right)=k_{\infty }\left[1-e^{-\frac{L}{{2L}_{C}}}\right] \end{array}$$

where (*k*_∞_) is the extrapolated asymptotic thermal conductivity and (*L_c_*) is a fitted characteristic normalized length. This relation was used as an empirical representation of the observed longitudinal size dependence and was not treated as a universal theoretical relation for dilute-gas transport.

**Figure 7:**
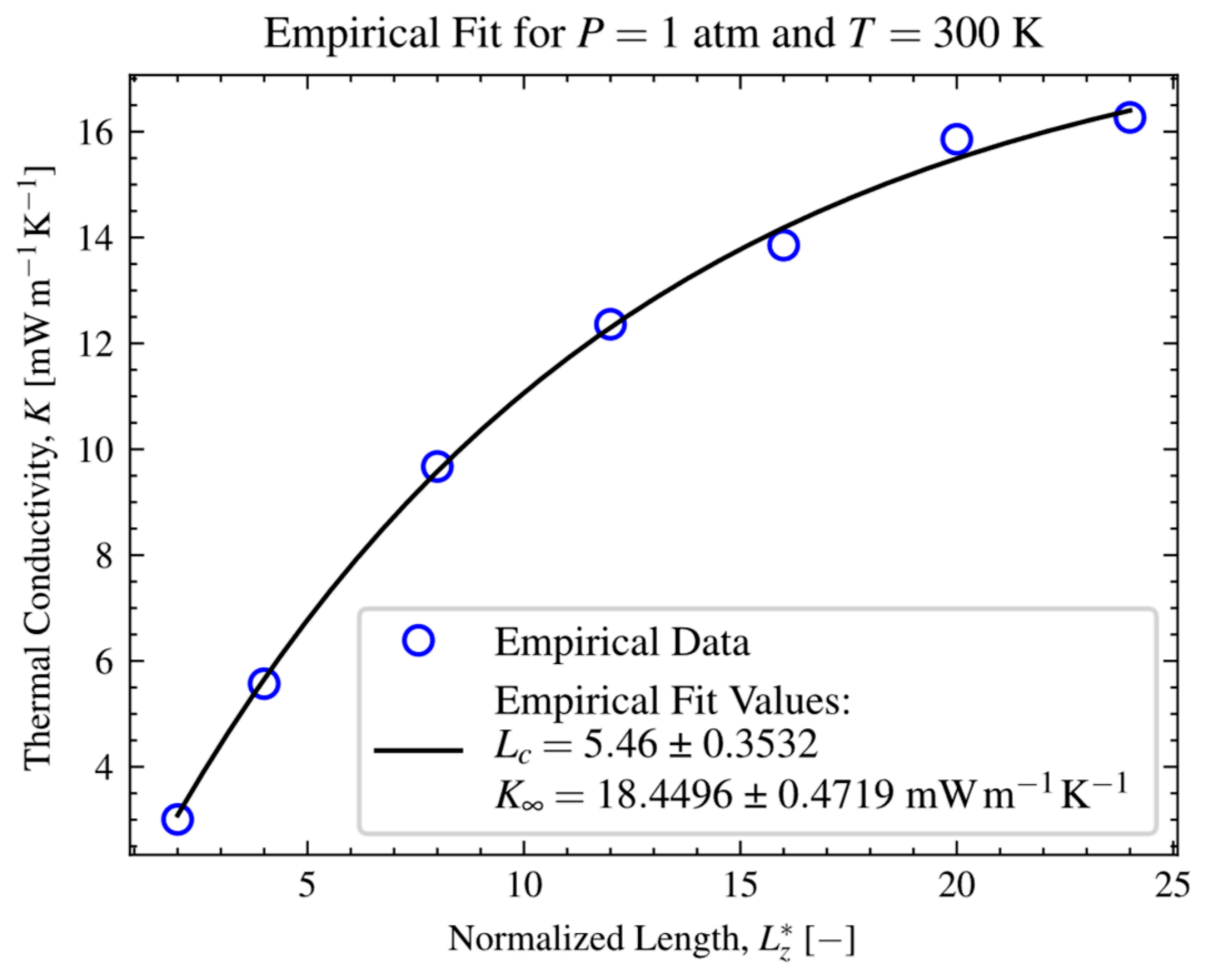
NEMD thermal conductivity as a function of normalized longitudinal system size for 1 atm. Symbols represent the simulation results, and lines represent the empirical fits.

Because the transverse dimensions remained fixed at (Lₓ* = L_y_* = 0.5), (*k*_∞_) represents the conductivity extrapolated with respect to the longitudinal dimension under the selected transverse dimensions. Complete three-dimensional bulk convergence would additionally require verification of transverse-size independence. The fitting was performed using the curve_fit routine in the SciPy Python library (version 1.13.1). Weighted least-squares regression was applied using

(20) $$\begin{array}{c} \sigma_{i}=\frac{1}{\sqrt{L}} \end{array}$$

which assigns greater weight to results obtained from larger longitudinal systems. Larger systems contain more atoms and generally provide smoother spatially averaged temperature profiles. The prescribed (*σ_i_*) values were used as fitting weights and were not interpreted as directly measured statistical uncertainties. The fitting procedure returned the optimized values of (*k*_∞_) and (*L_c_*), together with their covariance matrix. A simple fit-sensitivity analysis using alternative regression weights and fitting ranges were performed, which changed the extrapolated *k*_∞_ by less than 0.1 mW/m/K, indicating that the result is not strongly dependent on the fitting choice. The extrapolated conductivity values were then compared across the investigated pressures to determine whether the simulations recovered the expected pressure independence of bulk dilute-gas thermal conductivity. Figure 7 presents the calculated conductivity as a function of longitudinal system size for a given pressure, together with the corresponding empirical fit.

For all investigated pressures, the calculated conductivity increased as the longitudinal dimension increased. The rate of increase gradually decreased at larger values of (L_z_*), and the fitted curves approached asymptotic values. This behavior demonstrates that conductivity obtained from a finite NEMD domain depends on the longitudinal system size. A value calculated using one simulation length cannot automatically be treated as the large-length conductivity. When (L_z_) is comparable to the mean free path, molecules undergo relatively few collisions while transferring energy between the source and sink. Increasing (L_z_/λ) increases the opportunity for collision-driven energy redistribution and moves the simulated response toward its large-length limit. The empirical relation represented the overall trends reasonably well. Larger deviations were observed for some of the longest simulations at 0.2 atm. Fewer samples were collected for these cases because of their greater computational cost with the number of samples for each simulation shown in Table 3.

The extrapolated conductivity obtained at 0.2 atm is therefore less reliable than the values obtained at the higher pressures.

### 4.3. Recovery of Pressure-Independent Thermal Conductivity

**Figure 8:**
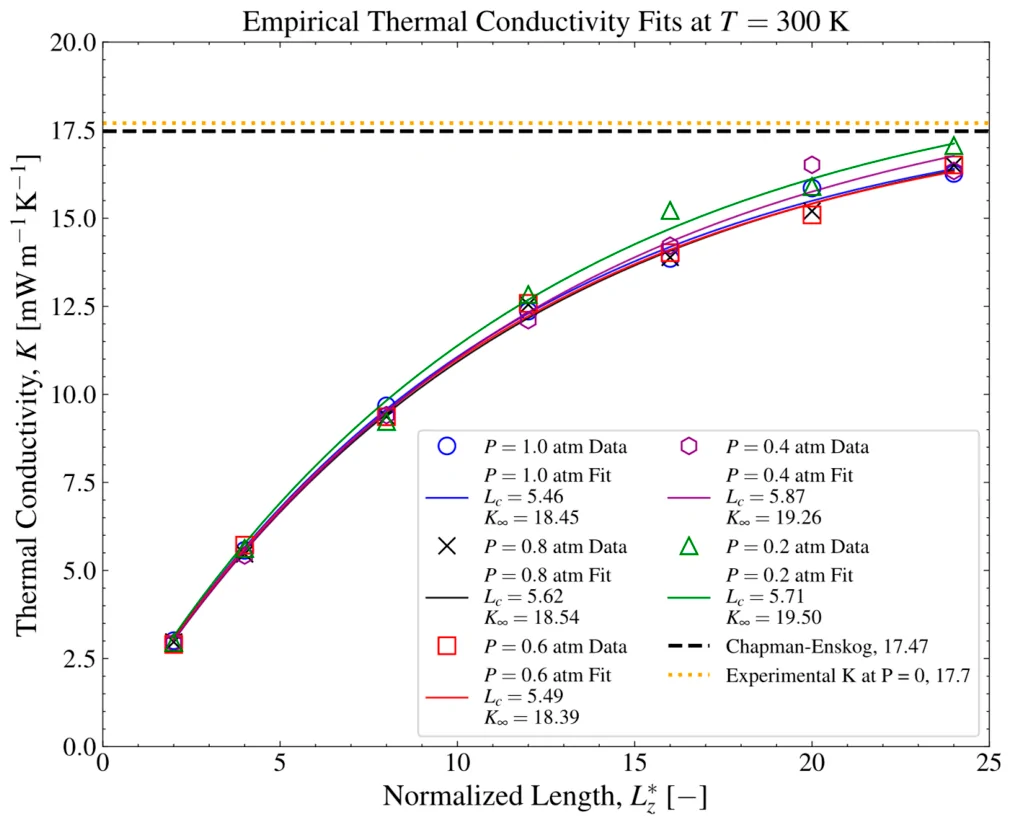
The combined comparison of the NEMD thermal-conductivity results and empirical fits at 300 K for pressures from 0.2 to 1.0 atm.

Figure 8 combines the NEMD conductivity results and empirical fits for all five investigated pressures. The simulation results and fitted curves remain close across the investigated pressures. The extrapolated conductivity values also show only small numerical variations across pressure except for the less reliable 0.2 atm result. This behavior is consistent with the kinetic-theory prediction that the thermal conductivity of a dilute gas is independent of pressure at fixed temperature. The simulations also show why finite MD calculations can appear to contradict this behavior. The target conductivity remains constant, but the molecular mean free path and the physical simulation length required to reach a given (*L_z_*/*λ*) increase as the pressure decreases.

The pressure comparison should therefore be based on conductivities evaluated over comparable normalized dimensions and examined for longitudinal size dependence. Comparing simulations with the same physical length at different pressures could instead compare different molecular transport regimes. The conductivity curves for the different pressures were located close to one another, particularly as the normalized longitudinal system size increased. The fitted asymptotic conductivity values also showed only small numerical variations across pressure except for the less reliable 0.2 atm result. The extrapolated conductivities remained above the reference value for argon at 300 K. Because only L_z_* was varied while Lₓ* = L_y_* = 0.5, the fits establish longitudinal convergence only. The fixed transverse dimensions may contribute to the remaining discrepancy, but this possibility was not tested fully since only three transverse simulations were performed to determine the likely dependence on the transverse direction. To assess this effect, simulations with transverse lengths Lₓ* = L_y_* = 0.5, 1, and 2 were performed at T = 300 K and P = 1 atm using a longitudinal length of L_z_* = 12. This value of L_z_* was selected because it is close to the fully developed thermal conductivity while remaining computationally efficient compared to larger longitudinal domains. The resulting thermal conductivities for the three transverse sizes were 12.36, 12.26, and 13.16 mW/m/K, respectively. These values exhibit small, scattered variations with no clear systematic trend, in contrast to the strong and monotonic finite-size effects observed along the heat-transfer direction. This suggests that the observed behavior is not primarily attributable to the transverse dimensions over the tested range. Nevertheless, additional simulations varying L_x_* and L_y_* are required before the extrapolated values can be considered fully converged bulk conductivities.

This behavior is consistent with the kinetic-theory expectation that the bulk thermal conductivity of a dilute monatomic gas is independent of pressure at fixed temperature. The finite-system conductivity nevertheless depended on longitudinal size because the mean free path and therefore the physical dimensions corresponding to the same normalized size changed with pressure. Thus, pressure-independent behavior was recovered only after the longitudinal size dependence was accounted for.

The NEMD results therefore distinguish between two separate effects:

1. The bulk thermal conductivity remains unchanged with pressure in the dilute-gas limit.
2. The physical simulation length required to approach that value increases because the molecular mean free path increases as pressure decreases.

This distinction explains why pressure-independent gas conductivity does not imply pressure-independent molecular dynamics simulation requirements.

Overall, the NEMD calculations recover the expected pressure-independent behavior of dilute-argon thermal conductivity after the longitudinal size dependence is accounted for. However, quantitative agreement with the reference conductivity requires further verification of transverse-size convergence and statistical uncertainty. Therefore, obtaining the correct pressure trend does not by itself establish that the absolute bulk conductivity has been fully converged.

### 4.4. EMD and NEMD Extrapolation Result Comparison

**Figure 9:**
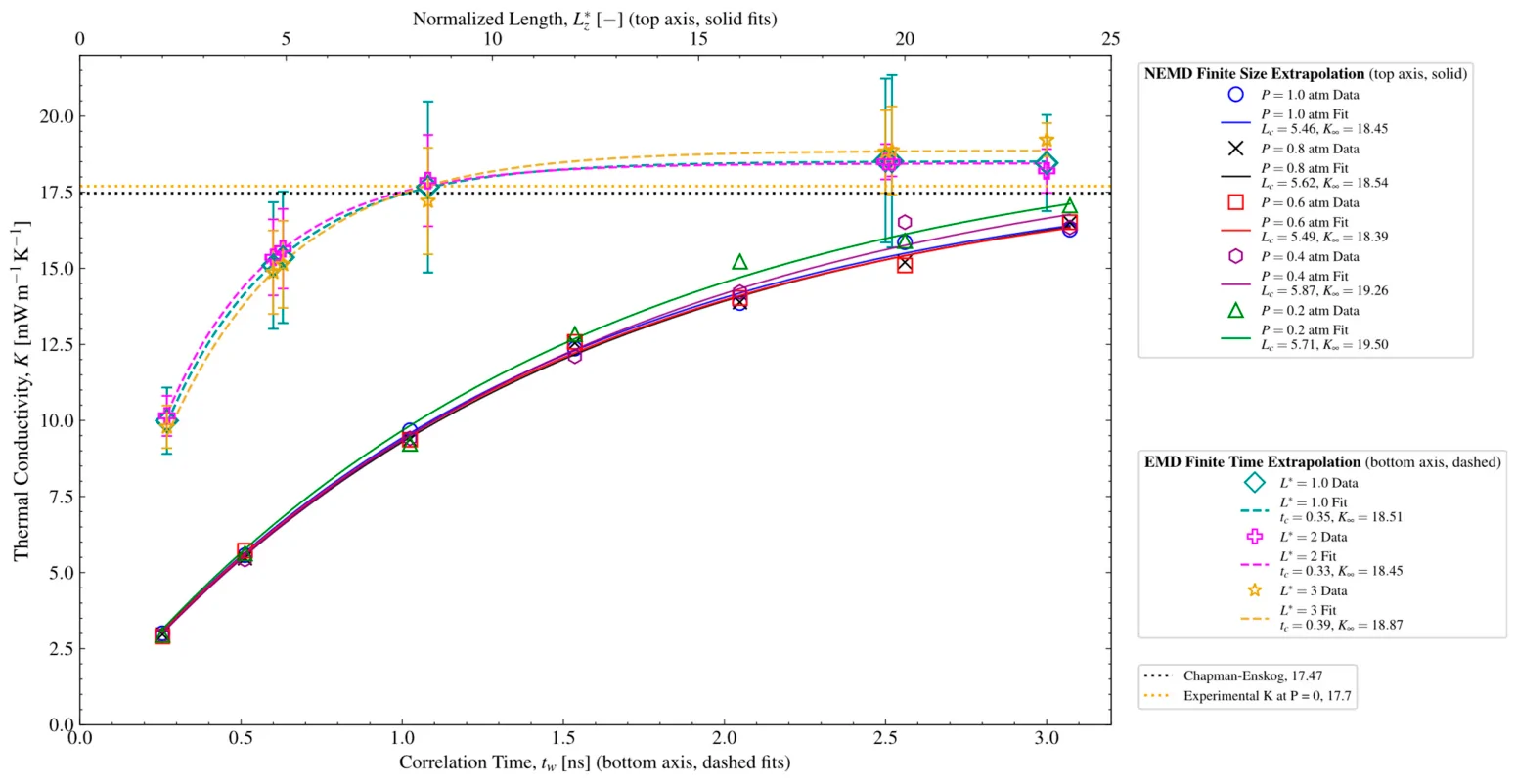
The combined comparison of EMD (dashed lines) and NEMD (solid lines) thermal-conductivity results for all investigated simulations. The EMD results use the primary *x*-axis only, while the NEMD results use the secondary *x*-axis only.

Figure 9 presents the EMD and NEMD conductivity results and their corresponding empirical fits on a shared *y*-axis. The bottom *x*-axis corresponds exclusively to the EMD sampling window T_w_, while the top *x*-axis corresponds exclusively to the NEMD system length L_z_*. These axes are independent, and no physical mapping between T_w_ and L_z_* are implied.

Across all cases, the extrapolated conductivities from both methods fall within 18–20 mW/m/K, indicating that independently obtained EMD and NEMD results are consistent with each other and with the bulk extrapolated value. However, this does not imply that the two methods converge to an identical bulk limit but rather that their fitted values fall within a similar range under the conditions studied.

## 5. Conclusions

This study examined the calculation of dilute-argon thermal conductivity using equilibrium and nonequilibrium molecular dynamics. The Green–Kubo calculations showed that different sampling intervals and correlation lengths produced similar conductivities when they resulted in similar correlation-window times. Increasing the correlation window initially moved the conductivity toward the expected value, while longer windows increased the scatter among the directional conductivities. Importantly, a conductivity close to the expected value could be obtained even when the running Green–Kubo integral had not clearly converged. Agreement with a reference value therefore does not by itself demonstrate convergence.

The NEMD calculations showed a clear dependence on the longitudinal simulation length. The calculated conductivity increased as the system length increased relative to the mean free path and gradually approached an asymptotic value. A conductivity obtained from one finite simulation domain cannot therefore be assumed to represent the bulk property, particularly when the system length is comparable to the molecular mean free path.

After the longitudinal size dependence was accounted for, the conductivity curves and fitted asymptotic values remained close across the investigated pressures. This recovers the kinetic-theory result that dilute-gas thermal conductivity is independent of pressure at a fixed temperature. At the same time, the physical simulation length required to approach this value increased as pressure decreased because the mean free path became larger. Pressure-independent conductivity therefore does not imply pressure-independent molecular dynamics simulation requirements.

The extrapolated conductivities remained above the reference value for argon at 300 K. Because only the longitudinal dimension was varied while the normalized transverse dimensions remained fixed at Lₓ* = L_y_* = 0.5, the present analysis establishes longitudinal convergence only. Additional simulations varying the transverse dimensions and providing improved statistical uncertainty estimates are needed before the absolute conductivity can be considered fully converged. Overall, the results demonstrate that reliable dilute-gas thermal conductivity calculations require careful examination of both the time scales used in Green–Kubo calculations and the length scales used in NEMD simulations.

## Figures and Tables

**Table 1:** The effect of Green–Kubo sampling and integration parameters on the calculated thermal conductivity of dilute argon.

Sample Interval (s)	Correlation Length (p)	L*	Pressure (atm)	Temperature (K)	T_w_ (ns)	k_x_ (W/m/k)	k_y_ (W/m/k)	k_z_ (W/m/k)	k_avg_ (W/m/k)	k_theory_ (W/m/k)
90	3000	1	1	300	0.27	0.00967 ± 0.00070	0.00979 ± 0.00109	0.01052 ± 0.00217	0.00999 ± 0.00109	0.0175
200	3000	1	1	300	0.6	0.01454 ± 0.00132	0.01455 ± 0.00213	0.01617 ± 0.00394	0.01509 ± 0.00208	0.0175
90	7000	1	1	300	0.63	0.01481 ± 0.00138	0.01479 ± 0.00220	0.01649 ± 0.00409	0.01536 ± 0.00216	0.0175
360	3000	1	1	300	1.08	0.01691 ± 0.00284	0.01649 ± 0.00258	0.01962 ± 0.00347	0.01767 ± 0.00281	0.0175
5000	500	1	1	300	2.5	0.01840 ± 0.00135	0.01743 ± 0.00399	0.01979 ± 0.00926	0.01854 ± 0.00269	0.0175
360	7000	1	1	300	2.52	0.01817 ± 0.00230	0.01644 ± 0.00177	0.02094 ± 0.00480	0.01852 ± 0.00283	0.0175
1000	3000	1	1	300	3	0.01837 ± 0.00132	0.01724 ± 0.00566	0.01978 ± 0.00769	0.01846 ± 0.00158	0.0175
90	3000	2	1	300	0.27	0.01065 ± 0.00179	0.00999 ± 0.00053	0.00981 ± 0.00035	0.01015 ± 0.00066	0.0175
200	3000	2	1	300	0.6	0.01618 ± 0.00414	0.01499 ± 0.00043	0.01491 ± 0.00101	0.01536 ± 0.00125	0.0175
90	7000	2	1	300	0.63	0.01646 ± 0.00434	0.01527 ± 0.00041	0.01518 ± 0.00111	0.01564 ± 0.00131	0.0175
360	3000	2	1	300	1.08	0.01945 ± 0.00268	0.01696 ± 0.00173	0.01724 ± 0.00285	0.01788 ± 0.00150	0.0175
5000	500	2	1	300	2.5	0.01846 ± 0.00484	0.01896 ± 0.00472	0.01807 ± 0.00437	0.01850 ± 0.00058	0.0175
360	7000	2	1	300	2.52	0.01844 ± 0.00463	0.01893 ± 0.00460	0.01810 ± 0.00424	0.01849 ± 0.00047	0.0175
1000	3000	2	1	300	3	0.01816 ± 0.00404	0.01841 ± 0.00547	0.01802 ± 0.00348	0.01820 ± 0.00072	0.0175
90	3000	3	1	300	0.27	0.00985 ± 0.00188	0.00997 ± 0.00095	0.00956 ± 0.00041	0.00979 ± 0.00070	0.0175
200	3000	3	1	300	0.6	0.01510 ± 0.00373	0.01540 ± 0.00219	0.01411 ± 0.00140	0.01487 ± 0.00137	0.0175
90	7000	3	1	300	0.63	0.01539 ± 0.00388	0.01567 ± 0.00228	0.01432 ± 0.00155	0.01513 ± 0.00143	0.0175
360	3000	3	1	300	1.08	0.01716 ± 0.00453	0.01766 ± 0.00457	0.01681 ± 0.00321	0.01721 ± 0.00175	0.0175
5000	500	3	1	300	2.5	0.01852 ± 0.00665	0.02034 ± 0.00622	0.01762 ± 0.00900	0.01883 ± 0.00136	0.0175
360	7000	3	1	300	2.52	0.01852 ± 0.00663	0.02035 ± 0.00664	0.01772 ± 0.00891	0.01887 ± 0.00145	0.0175
1000	3000	3	1	300	3	0.01900 ± 0.00639	0.02047 ± 0.00517	0.01818 ± 0.00957	0.01922 ± 0.00055	0.0175

**Table 2:** NEMD simulation characteristics for investigated pressures.

Pressure (atm)	Temperature (K)	Non-Dimensional Simulation Dimensions					Simulation Dimensions					Atoms
		L_x_*	L_y_*	L_z_*	L_sink_*	L_source_*	L_x_ (Å)	L_y_ (Å)	L_z_ (Å)	L_sink_ (Å)	L_source_ (Å)	
1	300	0.5	0.5	2	0.1	0.1	343.4242	343.4242	1373.6968	68.68484	68.68484	3963
1	300	0.5	0.5	4	0.2	0.2	343.4242	343.4242	2747.3936	137.36968	137.36968	7927
1	300	0.5	0.5	8	0.4	0.4	343.4242	343.4242	5494.7871	274.739355	274.739355	15,854
1	300	0.5	0.5	12	0.6	0.6	343.4242	343.4242	8242.1807	412.109035	412.109035	23,781
1	300	0.5	0.5	16	0.8	0.8	343.4242	343.4242	10,989.574	549.4787	549.4787	31,708
1	300	0.5	0.5	20	1	1	343.4242	343.4242	13,736.968	686.8484	686.8484	39,635
1	300	0.5	0.5	24	1.2	1.2	343.4242	343.4242	16,484.361	824.21805	824.21805	47,562
0.8	300	0.5	0.5	2	0.1	0.1	429.2802	429.2802	1717.121	85.85605	85.85605	6193
0.8	300	0.5	0.5	4	0.2	0.2	429.2802	429.2802	3434.2419	171.712095	171.712095	12,386
0.8	300	0.5	0.5	8	0.4	0.4	429.2802	429.2802	6868.4839	343.424195	343.424195	24,772
0.8	300	0.5	0.5	12	0.6	0.6	429.2802	429.2802	10,302.726	515.1363	515.1363	37,158
0.8	300	0.5	0.5	16	0.8	0.8	429.2802	429.2802	13,736.698	686.8349	686.8349	49,544
0.8	300	0.5	0.5	20	1	1	429.2802	429.2802	17,171.21	858.5605	858.5605	61,930
0.8	300	0.5	0.5	24	1.2	1.2	429.2802	429.2802	20,605.452	1030.2726	1030.2726	74,316
0.6	300	0.5	0.5	2	0.1	0.1	572.3737	572.3737	2289.4946	114.47473	114.47473	11,010
0.6	300	0.5	0.5	4	0.2	0.2	572.3737	572.3737	4578.9893	228.949465	228.949465	22,019
0.6	300	0.5	0.5	8	0.4	0.4	572.3737	572.3737	9157.9785	457.898925	457.898925	44,039
0.6	300	0.5	0.5	12	0.6	0.6	572.3737	572.3737	13,736.968	686.8484	686.8484	66,058
0.6	300	0.5	0.5	16	0.8	0.8	572.3737	572.3737	18,315.957	915.79785	915.79785	88,078
0.6	300	0.5	0.5	20	1	1	572.3737	572.3737	22,894.946	1144.7473	1144.7473	110,097
0.6	300	0.5	0.5	24	1.2	1.2	572.3737	572.3737	27,473.936	1373.6968	1373.6968	132,117
0.4	300	0.5	0.5	2	0.1	0.1	858.5605	858.5605	3434.2419	171.712095	171.712095	24,772
0.4	300	0.5	0.5	4	0.2	0.2	858.5605	858.5605	6868.4839	343.424195	343.424195	49,544
0.4	300	0.5	0.5	8	0.4	0.4	858.5605	858.5605	13,736.968	686.8484	686.8484	99,087
0.4	300	0.5	0.5	12	0.6	0.6	858.5605	858.5605	20,605.452	1030.2726	1030.2726	148,631
0.4	300	0.5	0.5	16	0.8	0.8	858.5605	858.5605	27,473.936	1373.6968	1373.6968	198,175
0.4	300	0.5	0.5	20	1	1	858.5605	858.5605	34,342.419	1717.12095	1717.12095	247,719
0.4	300	0.5	0.5	24	1.2	1.2	858.5605	858.5605	41,210.903	2060.54515	2060.54515	297,262
0.2	300	0.5	0.5	2	0.1	0.1	1717.121	1717.121	6868.4839	343.424195	343.424195	99,087
0.2	300	0.5	0.5	4	0.2	0.2	1717.121	1717.121	13,736.968	686.8484	686.8484	198,175
0.2	300	0.5	0.5	8	0.4	0.4	1717.121	1717.121	27,473.936	1373.6968	1373.6968	396,360
0.2	300	0.5	0.5	12	0.6	0.6	1717.121	1717.121	41,210.903	2060.54515	2060.54515	594,525
0.2	300	0.5	0.5	16	0.8	0.8	1717.121	1717.121	54,947.871	2747.39355	2747.39355	792,700
0.2	300	0.5	0.5	20	1	1	1717.121	1717.121	68,684.834	3434.2417	3434.2417	990,875
0.2	300	0.5	0.5	24	1.2	1.2	1717.121	1717.121	82,421.807	4121.09035	4121.09035	1,189,050

**Table 3:** The number of samples per simulations as L_z_* increased from 2 to 24 for the 0.2 atm case.

L_z_*	Samples
2	800
4	800
8	792
12	459
16	335
20	208
24	176

## Data Availability

The raw data supporting the conclusions of this article will be made available by the authors on request.
